# Modeling Neurodegeneration *in silico* With Deep Learning

**DOI:** 10.3389/fninf.2021.748370

**Published:** 2021-11-19

**Authors:** Anup Tuladhar, Jasmine A. Moore, Zahinoor Ismail, Nils D. Forkert

**Affiliations:** ^1^Department of Radiology, University of Calgary, Calgary, AB, Canada; ^2^Hotchkiss Brain Institute, University of Calgary, Calgary, AB, Canada; ^3^Biomedical Engineering Program, University of Calgary, Calgary, AB, Canada; ^4^Department of Clinical Neurosciences, University of Calgary, Calgary, AB, Canada; ^5^Department of Community Health Sciences, University of Calgary, Calgary, AB, Canada; ^6^Department of Psychiatry, University of Calgary, Calgary, AB, Canada; ^7^O’Brien Institute for Public Health, University of Calgary, Calgary, AB, Canada; ^8^Alberta Children’s Hospital Research Institute, University of Calgary, Calgary, AB, Canada

**Keywords:** deep neural networks (DNN), posterior cortical atrophy (PCA), neurodegenerative diseases, Alzheimer’s disease, *in silico* simulation, visual object recognition, visual object agnosia, cognitive computational neuroscience

## Abstract

Deep neural networks, inspired by information processing in the brain, can achieve human-like performance for various tasks. However, research efforts to use these networks as models of the brain have primarily focused on modeling healthy brain function so far. In this work, we propose a paradigm for modeling neural diseases *in silico* with deep learning and demonstrate its use in modeling posterior cortical atrophy (PCA), an atypical form of Alzheimer’s disease affecting the visual cortex. We simulated PCA in deep convolutional neural networks (DCNNs) trained for visual object recognition by randomly injuring connections between artificial neurons. Results showed that injured networks progressively lost their object recognition capability. Simulated PCA impacted learned representations hierarchically, as networks lost object-level representations before category-level representations. Incorporating this paradigm in computational neuroscience will be essential for developing *in silico* models of the brain and neurological diseases. The paradigm can be expanded to incorporate elements of neural plasticity and to other cognitive domains such as motor control, auditory cognition, language processing, and decision making.

## Introduction

By mimicking information processing and cognition in the human and primate brain (Kriegeskorte, 2015; Yamins and DiCarlo, 2016; Richards et al., 2019), deep neural networks have been shown to be capable of outperforming conventional machine learning methods for various classification and regression tasks, such as visual object recognition (LeCun et al., 2015; Vercio et al., 2020). Cognitive neuroscience continues to influence and advance the development of more biologically informed deep neural networks to further improve classification performance and achieve more human-like results (Kriegeskorte and Douglas, 2018). However, the potential for using deep neural networks as computational models of the human brain to advance our understanding of neurological diseases, such as Alzheimer’s disease, has been largely neglected (Khatami et al., 2020).

**FIGURE 1:**
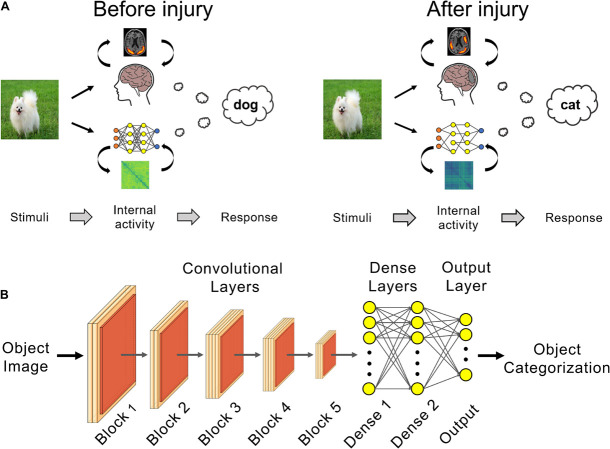
Deep learning can be used as *in silico* models of brain injury. **(A)** Changes in internal patterns of activity between neurons and in external behavior can be studied in trained networks before and after a simulated neural injury, such as for posterior cortical atrophy (PCA) as shown here. **(B)** VGG 19 networks trained for visual object recognition on the CIFAR-100 dataset were used for PCA simulation experiments.

In this work, we propose a paradigm using deep neural networks as *in silico* models of neurodegeneration (Figure 1A). Within this context, *in silico* refers to employing computer models to improve our understanding of normal and pathological processes in the living organism. To date, in the context of using deep neural networks as models of normal cognitive function, this has been mostly studied using deep convolutional neural networks (DCNNs) with a focus on visual cognition (Cadieu et al., 2014; Khaligh-Razavi and Kriegeskorte, 2014; Yamins et al., 2014; Hong et al., 2016; Kheradpisheh et al., 2016; Horikawa and Kamitani, 2017; Wen et al., 2018; Grossman et al., 2019; Horikawa et al., 2019). The connectivity and hierarchical organization of DCNNs are inspired by the mammalian visual cortex, which make them promising biomimetic models for this purpose (Kriegeskorte, 2015; Yamins and DiCarlo, 2016; Peters and Kriegeskorte, 2021). DCNNs are state-of-the-art models for the prediction of neural responses of the ventral visual stream, particularly in higher order regions such as visual area V4 and inferior temporal cortex, during visual object recognition (Cadieu et al., 2014; Yamins et al., 2014). Additionally, it has been shown that the internal representation of objects (Cadieu et al., 2014; Khaligh-Razavi and Kriegeskorte, 2014) and faces (Grossman et al., 2019) in DCNNs are similar to the internal representations in humans. Consequently, current efforts aim to quantify the similarity of different DCNN architectures to the human brain through integrative benchmarking, such as the Brain-Score (Schrimpf et al., 2020).

Prior work in modeling neurological diseases have primarily explored computational models of cellular processes focusing on pathological changes such as excitotoxicity or abnormal bioenergetics (Le Masson et al., 2014; Muddapu et al., 2019), or connectome models based on structural or functional connectivity in the brain (Hof et al., 1997; Raj et al., 2012; Zhou et al., 2012; Ortiz et al., 2015; Peraza-Goicolea et al., 2020; Vanasse et al., 2021). Early work investigating structural or functional connectomes primarily focused on modeling specific aspects of disease progression, such as diffusive spread of misfolded tau and beta amyloids (Raj et al., 2012) or changes in network connectivity contributing to disease vulnerability or diagnosis (Zhou et al., 2012; Ortiz et al., 2015). More recent work has begun using the connectome to simulate disease states *in silico*, for example by modifying connection weights in a simulated functional connectome to predict changes in functional activation and connectivity in the brain (Peraza-Goicolea et al., 2020). A meta-analysis of the relationship between structural pathology and behavioral pathology supported the notion that network degeneration is a contributing factor to disease pathology (Vanasse et al., 2021). However, none of the aforementioned approaches used models that can perform tasks at near-human levels (LeCun et al., 2015; Vercio et al., 2020). Doing so would enable the simulation of changes in model behavior with disease progression *in silico*.

Deep neural network-based approaches have largely focused on data-driven analysis (Khatami et al., 2020), such as disease diagnosis or progression prediction (Lee et al., 2019; Martinez-Murcia et al., 2019). One notable exception is the work by Lusch et al. (2018) who used a DCNN to model traumatic brain injury (TBI) as focal axonal swellings that cause spike train blockage. In these analyses, TBI was simulated by ablating DCNN weights. The work by Lusch et al. (2018) focused primarily on abruptly injuring the DCNN, with a very limited investigation of cumulative network injury, and did not quantify changes in artificial neuron activation patterns or representations. Additionally, the similarity of the DCNNs used in the study to biological neural networks, and thus their validity as brain-like models of visual cognition, were not investigated (Schrimpf et al., 2020).

In this study, we used DCNNs trained for visual object recognition to model posterior cortical atrophy (PCA), an atypical form of Alzheimer’s marked by lesions in the visual cortex and attendant visual cognition deficits such as visual agnosia (Tang-Wai et al., 2004; Crutch et al., 2012; Migliaccio et al., 2016; Silva et al., 2017). Specifically, we trained the VGG-19 DCNN (Simonyan and Zisserman, 2014) to perform visual object recognition on the CIFAR-100 dataset (Krizhevsky, 2009). VGG-19 has one of the highest similarities to biological neural networks amongst feed-forward DCNNs as measured by the Brain-Score (Schrimpf et al., 2020). Trained networks underwent neurodegeneration, where connections between artificial neurons, mimicking biological synapses, were progressively lesioned. At each stage of simulated PCA we evaluated the respective changes in the injured network’s object classification performance and activation patterns of its neurons.

## Materials and Methods

### Dataset and Model Training

We trained the VGG-19 DCNN (Simonyan and Zisserman, 2014) to perform visual object recognition on the CIFAR-100 dataset (Krizhevsky, 2009). CIFAR-100 is a publicly available natural images dataset which contains 50,000 training and 10,000 testing images (32 × 32 pixels) of objects from 100 classes (e.g., roses, butterflies, and bicycles) organized into 20 categorical superclasses (e.g., flowers, insects, and vehicles).

The VGG-19 DCNN has 16 convolutional layers followed by two fully-connected dense layers with 1,000 neurons each (Figure 1B). The network’s output layer has 100 neurons and classifies the input image into one of the 100 classes using the softmax function. The convolutional layers were initialized with weights pretrained on ImageNet (Deng et al., 2009) in order to make use of the convolutional kernel features learned on this much larger database. Input images were upscaled 4× to 128 × 128 pixels and the fully-connected dense and output layers were randomly initialized.

Dropout regularization (30%) was used after each fully-connected dense layer (“Dense 1” and “Dense 2,” Figure 1B). This regularization technique helps reduce network overfitting to the training data and improve generalization performance of the uninjured baseline networks (Srivastava et al., 2014). Briefly, in each training round, 30% of the neurons and associated weights in the fully-connected dense layers were temporarily removed during training. The neurons and associated weights are restored after the training round and in the final, fully-trained, baseline uninjured model.

Training parameters resulting in the best network performance were optimized for the final model in preliminary investigations. More precisely, a fixed learning rate of 3 × 10^–5^ was used with the RMSprop optimizer for 40 training epochs. The network was trained end-to-end, such that both the pretrained convolutional layers and the randomly initialized fully-connected dense and output layers were updated during training. This training was repeated with different random initializations of the fully-connected dense and output classification layers to produce 25 unique uninjured networks for use in subsequent experiments on simulating PCA injury. The uninjured networks produced at the end of training were used in these experiments regardless of training performances. Though no exclusion criterion was applied to uninjured networks after training, the use of 25 unique networks reduces potential bias from single outliers. The uninjured networks achieved an average test set performance of 76.48% (standard deviation: 0.53%). Importantly, the networks were only given the class labels during training and no information about an object’s superclass.

### Simulating Posterior Cortical Atrophy

The trained networks underwent simulated PCA injury by randomly setting x% of the network weights to zero, effectively severing the connections between artificial neurons. This was done for trained weights between all layers in a network, i.e., the weights between all the convolutional layers, dense layers and output layer (Figure 1B). We simulated the visual agnosia seen in PCA by repeatedly injuring each network at 0.1% increments such that the damage was cumulative. In other words, for each incremental injury, the previous injured connections remained at zero and an additional set of randomly selected connections are set to zero. The selection of weights to injure was random for each of the 25 trained networks, such that the course of degeneration was unique for each of the 25 networks.

### Quantifying Changes in Network Behavior

After each injury, networks’ accuracy in visual object recognition was measured on the set of test images. Random chance performance on visual object recognition was determined by calculating the probability of randomly selecting the correct class out of the 100 classes. As the number of test examples in each class is equal (100), the random chance performance is 1%.

The network errors were further analyzed by evaluating the percentage of misclassified examples that were in the correct superclass, e.g., misidentifying a rose as another type of flower such as a tulip. This analysis was done on the test examples that the network made an incorrect prediction on. The incorrectly predicted class label was converted to the corresponding superclass label. This converted superclass label is an indirectly predicted superclass label, as the networks were never given information about the class-superclass structure of the data. The converted superclass label is compared to the true superclass label to determine if the network’s incorrectly predicted class was within the correct superclass. This was calculated over all test set examples to obtain the percentage of errors that were within the correct superclass. The average level of errors within the correct superclass for the 25 baseline uninjured networks was 39.37% (standard deviation: 0.75%). Random chance for network errors within the correct superclass was calculated based on the probability of selecting the correct superclass out of the 20 superclasses. As the number of test examples in each superclass is equal (500), the random chance performance is 5%.

Representational similarity analysis (Kriegeskorte et al., 2008) was used to quantify changes in the learned internal representations of visual objects over the course of simulated PCA. Activations from neurons in a network’s penultimate layer (“Dense 2,” Figure 1B) were compared for pairs of test set visual stimuli using 1 – Pearson’s correlation. This was done over all possible pairs of stimuli in the test set to create the representational dissimilarity matrices (RDMs), which provides an overview of a network’s learned representation (Kriegeskorte et al., 2008; Khaligh-Razavi and Kriegeskorte, 2014; Yamins et al., 2014). The changes in a network’s internal representation with injury was calculated by comparing the correlation of an injured network’s RDM to the same model’s uninjured RDM using Kendall’s τ_A_ correlation coefficient. Random noise levels for Kendall’s τ_A_ correlation coefficient were calculated as the Kendall’s τ_A_ of an uninjured network’s RDM compared to a scrambled version of the same uninjured network’s RDM. This was averaged across the uninjured RDMs for the 25 trained uninjured networks to obtain the random noise level of 0.005% for Kendall’s τ_A_.

### Statistical Analysis

Object recognition performances, RDMs, and Kendall’s τ_A_ correlation coefficients were averaged across the 25 trained networks. Results were analyzed using PRISM (GraphPad, v9.0). Statistical significance was reported when *P* < 0.01 and was determined using one-way repeated measures ANOVA or two-way ANOVA and Bonferroni’s *post hoc* test for multiple comparisons, as appropriate.

## Results

**FIGURE 2:**
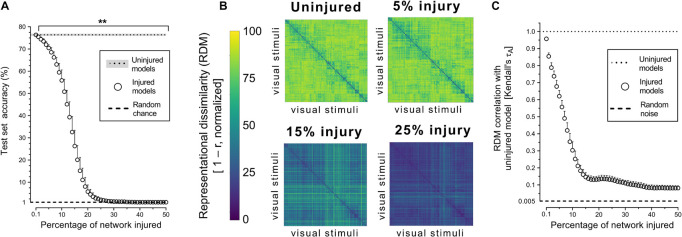
Trained VGG-19 networks underwent progressive neurodegeneration. **(A)** Object recognition performance of injured VGG-19 models on the CIFAR-100 dataset with simulated PCA. **(B)** Internal activity of artificial neurons in the model’s penultimate layer visualized with representational similarity analysis. The RDMs visualized are the average of the 25 trained networks. **(C)** Changes in internal activity patterns relative to uninjured control models measured using Kendall’s τ_A_ correlation coefficient. Data are shown as mean + SD, *n* = 25 models, **P < 0.01 with one-way repeated measures ANOVA with Bonferroni’s correction for multiple comparisons.

Object recognition performance was already impaired by simulated PCA injury of just 0.2% of connections (Figure 2A). By 30% injury, the network performance was reduced to chance level (1%). RDMs, used to analyze object representations within degenerating networks, showed that representations became more homogenous with injury (Figure 2B). Though object-level representations deteriorated markedly with injury, the networks retained some structured internal representations (Figure 2C). Even when networks lost all object recognition capability after 30% injury, the deteriorated internal representations retained correlation with the representations of uninjured networks and remained above the level of random noise (0.005%).

**FIGURE 3:**
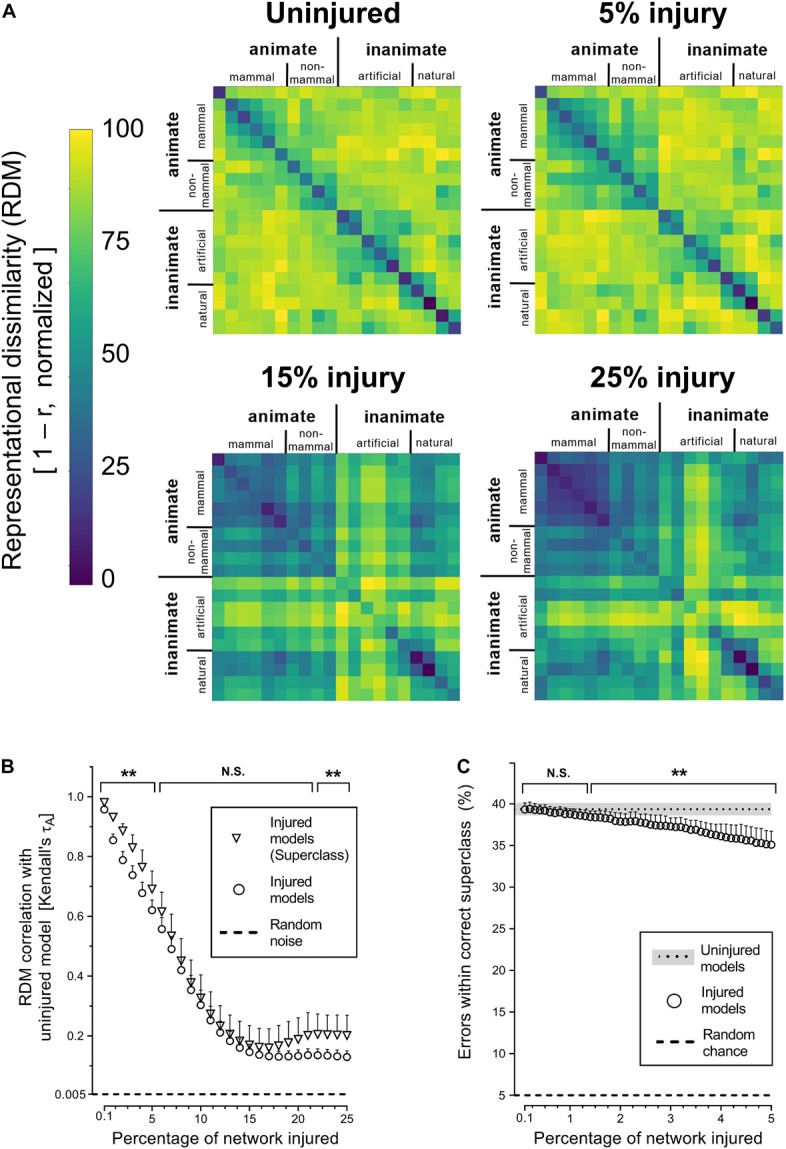
Degenerating neural networks retained generalized information better than specific details. **(A)** Representational similarity of objects within a categorical superclass. The RDMs visualized are the average of the 25 trained networks. **(B)** Changes in internal activity patterns, relative to uninjured control models, at the object and superclass-level measured using Kendall’s τ_A_ correlation coefficient. **(C)** Percentage of misclassified objects that were within the correct categorical superclass. Data are shown as mean + SD, *n* = 25 models, **P < 0.01 using two-way ANOVA **(B)** or one-way repeated measures ANOVA **(C)** with Bonferroni’s correction for multiple comparisons.

Next, we investigated whether simulated PCA impacted the networks’ representational capacity at all levels of semantic hierarchy equally by analyzing superclass-level RDMs. The corresponding results showed that trained networks were generally able to infer the existence of the categorical superclasses in the CIFAR-100 dataset, despite never have been given this information explicitly (Figure 3A). The internal representations of categorical superclass and broader image categories, such as animate vs. inanimate or natural vs. artificial, were better retained with injury compared to object-level representations (Figures 3A,B). This increased stability of superclass representation was also reflected in the manner in which networks made mistakes at the initial stages of simulated PCA, as objects were misclassified for another object within the same superclass (e.g., a rose is misclassified as another type of flower) at the same rate as uninjured networks until 1.3% injury; thereafter, the networks made more random errors (Figure 3C).

## Discussion

Deep learning is a powerful biomimetic paradigm for modeling cognitive processes and information processing in the brain (Richards et al., 2019). Efforts thus far on using deep learning to study the human brain have primarily focused on modeling normal function (Cadieu et al., 2014; Khaligh-Razavi and Kriegeskorte, 2014; Yamins et al., 2014; Hong et al., 2016; Kheradpisheh et al., 2016; Horikawa and Kamitani, 2017; Kell et al., 2018; Pandarinath et al., 2018; Wen et al., 2018; Grossman et al., 2019; Horikawa et al., 2019; Botvinick et al., 2020; Hashemzadeh et al., 2020; Caucheteux et al., 2021). In this paper, we present an important new direction, namely using deep learning for modeling brain dysfunction *in silico*. As deep neural networks incorporate neurally mechanistic features to increase their biological plausibility, it will be important to investigate how well they model both normal function and disease if they are to become truly brain-like (Kriegeskorte, 2015; Yamins and DiCarlo, 2016; Kriegeskorte and Douglas, 2018; Richards et al., 2019).

Based on the simulated PCA in a DCNN, it was shown that increasing functional deficits were associated with greater levels of injury in our artificial neural networks, parallel to increased visual agnosia with greater atrophy (Hof et al., 1997; Fox et al., 1999; Zarei et al., 2013; Silva et al., 2017). At the initial stages of simulated PCA, the injured networks tended to misclassify objects as another similar item, akin to misidentifying a fork as a knife by patients with visual agnosia (Milner et al., 1991). Simulated neurodegeneration affected object-level representations more severely than broader categorical-representations, similar to the loss of object-specific knowledge occurring prior to object category knowledge loss in patients with semantic dementia (Hodges et al., 1995).

This study is a first step in investigating the use of deep neural networks as *in silico* models of neural injury. As such, limitations on the current work and avenues for future investigations must be highlighted. Notably, the *in silico* model was more sensitive to injury compared to the human brain, showing measurable impairments after just 0.2% injury. In contrast, cognitive impairments do not typically manifest until much greater levels of cortical atrophy (Fox et al., 1999; Zarei et al., 2013). However, this must also take into consideration that clinical detection of PCA often occurs late in the disease course. PCA is easily misdiagnosed, with visual impairment interpreted as ophthalmologic or refractive problems, and cortical dysfunction considered only when cognitive impairment is more overt, resulting in accurate assessment and treatment (Crutch et al., 2012). Although a disease time course for PCA is not available, Lehmann et al. (2011) reported a 10–20% decrease of cortical thickness in the occipitoparietal and occipitotemporal regions of patients diagnosed with PCA (Lehmann et al., 2011), with gray matter loss of approximately 2% of whole brain volume per year (Lehmann et al., 2012). PCA patients show a range of cognitive deficits, with one study reporting a 40–50% decrease in visual task performance compared to controls (McMonagle et al., 2006). This magnitude of deficit is comparable with the decrease in performance with 10–20% injury in our model, though more rigorous comparison between deficits in clinical and simulated PCA are needed.

Another limitation is that our simulation assumed a static system that does not change its connectivity over the course of PCA. In contrast, the brain is a dynamic system that undergoes synaptic plasticity and functional connectivity changes in response to the insult itself, as well as to rehabilitative and pharmacologic interventions (Voss et al., 2017). This plasticity could be added to our *in silico* model by retraining the damaged DCNNs in between inflicting injuries. While this is outside the scope of this preliminary study, research efforts on DCNN pruning may provide some insight on injured network retraining. Pruning is often undertaken to compress networks by selectively removing weights to reduce the size and computational demand of the networks, for example by removing low magnitude weights (Han et al., 2015). However, with sufficient pruning, the networks will eventually suffer large declines in performance. To mitigate this, the networks can be retrained, such as after pruning or over the course of progressive pruning (Mittal et al., 2019; Marcin and Maciej, 2020). It has been shown that this retraining allows the removal of a substantially larger number of connections while retaining comparable performance. Applying these retraining algorithms to the injured networks may provide a way to mimic neuroplasticity *in silico*, which will be essential to capturing the complexities of neural injury in more biologically plausible models.

Moreover, the current study used weight ablation to simulate progressive neurodegeneration in a DCNN. However, it remains to be seen if alternative injury types may be more appropriate for simulating PCA or other neurodegenerative diseases. The network weight ablation injury used here simulates synaptic injury, whereas cortical atrophy seen in PCA affects both neurons and synapses (Lehmann et al., 2011, 2012). Thus, alternative approaches to modeling could include network node ablation to simulate neuronal injury, or a combination of weight and network node ablation to simulate a combination of synaptic and neuronal injury. Furthermore, alternative network modifications, such as randomly rewiring network nodes or setting network weights to random values instead of zero (Gaier and Ha, 2019; Xie et al., 2019), may also be studied to determine their appropriateness for modeling PCA or other types of dysfunction seen in neurodegenerative and neurological diseases.

Alternative courses of simulating the progressive injury seen in PCA and other neurodegenerative diseases may also be explored. For example, the current study used a sudden ablation injury of randomly selected weights across the entire network, with progressive injury simulated by increasing the number of ablated weights. An alternative approach could be to progressively decrease injured synapses to zero or add increasing noise to the weights. However, it remains to be seen whether this better recapitulates the slower manifestation of cognitive deficits seen clinically (Fox et al., 1999; Zarei et al., 2013). In contrast to random weight ablation, ablation of connections based on their strengths such as in network pruning (Han et al., 2015; Mittal et al., 2019; Marcin and Maciej, 2020), or specifically targeting excitatory (positive) or inhibitory (negative) connections (Song et al., 2016; Mackwood et al., 2021) may be instructive.

Future work may also investigate more biologically informed methods for modeling disease. One possibility may be to model the diffusive spread of disease-causing agents such as misfolded tau and beta amyloids across artificial neurons and synapses in the deep neural network (Raj et al., 2012; Vogel et al., 2021). Another approach may be to injure individual DCNN layers based on their correspondence to regions of the ventral visual stream such as V4 and inferior temporal cortex (Cadieu et al., 2014; Yamins et al., 2014). This layer-wise injury could be informed by neuroimaging, such as patterns of atrophy in PCA (Whitwell et al., 2007; Lehmann et al., 2011, 2012).

Finally, this paradigm of simulating neural injury can be extended to deep neural networks in other cognitive domains such as motor control (Pandarinath et al., 2018), auditory cognition (Kell et al., 2018), language processing (Hashemzadeh et al., 2020; Caucheteux et al., 2021), memory (Schapiro et al., 2017), and decision making (Botvinick et al., 2020). This may enable the development of *in silico* models of other neurological diseases, such as Parkinson’s disease, and the study of their impact on multiple cognitive systems.

In conclusion, our results show that deep neural networks may serve as *in silico* models of neurodegeneration, as the injured network’s behavior is similar to that seen clinically. Future work will need to study this correspondence with clinical data in more detail. Marrying *in silico* disease modeling with clinical data may enable the creation of patient-specific computational models, and may be integral to precision medicine for neurological diseases.

## Data Availability Statement

The CIFAR-100 dataset analyzed for this study is publicly available at https://www.cs.toronto.edu/~kriz/cifar.html. The pretrained VGG-19 used for this study is available from Tensorflow 2.0. The source code for fine-tuning pretrained VGG-19 models on CIFAR-100, for injuring models, and for evaluating their performance and RDMs are available from the corresponding author upon request.

## Author Contributions

AT, JM, and NF designed the study. AT and JM developed the computer code. AT performed the research, data analysis and drafted the manuscript. All authors revised the manuscript and approved the submitted version.

## Conflict of Interest

The authors declare that the research was conducted in the absence of any commercial or financial relationships that could be construed as a potential conflict of interest.

## Publisher’s Note

All claims expressed in this article are solely those of the authors and do not necessarily represent those of their affiliated organizations, or those of the publisher, the editors and the reviewers. Any product that may be evaluated in this article, or claim that may be made by its manufacturer, is not guaranteed or endorsed by the publisher.
